# Large cation ethylammonium incorporated perovskite for efficient and spectra stable blue light-emitting diodes

**DOI:** 10.1038/s41467-020-17943-6

**Published:** 2020-08-20

**Authors:** Zema Chu, Yang Zhao, Fei Ma, Cai-Xin Zhang, Huixiong Deng, Feng Gao, Qiufeng Ye, Junhua Meng, Zhigang Yin, Xingwang Zhang, Jingbi You

**Affiliations:** 1 Key Laboratory of Semiconductor Materials Science, Institute of Semiconductors, Chinese Academy of Sciences, Beijing, 100083 P. R. China; 2 Center of Materials Science and Optoelectronics Engineering, University of Chinese Academy of Sciences, Beijing, 100049 P. R. China; 3 State Key Lab Superlattices & Microstruct, Beijing 100083, Institute of Semiconductors, Chinese Academy of Sciences, Beijing, 100083 P. R. China

**Keywords:** Electronic devices, Organic LEDs

## Abstract

Perovskite light-emitting diodes (PeLEDs) have showed significant progress in recent years; the external quantum efficiency (EQE) of electroluminescence in green and red regions has exceeded 20%, but the efficiency in blue lags far behind. Here, a large cation CH_3_CH_2_NH_2_^+^ is added in PEA_2_(CsPbBr_3_)_2_PbBr_4_ perovskite to decrease the Pb–Br orbit coupling and increase the bandgap for blue emission. X-ray diffraction and nuclear magnetic resonance results confirmed that the EA has successfully replaced Cs^+^ cations to form PEA_2_(Cs_1-*x*_EA_*x*_PbBr_3_)_2_PbBr_4_. This method modulates the photoluminescence from the green region (508 nm) into blue (466 nm), and over 70% photoluminescence quantum yield in blue is obtained. In addition, the emission spectra is stable under light and thermal stress. With configuration of PeLEDs with 60% EABr, as high as 12.1% EQE of sky-blue electroluminescence located at 488 nm has been demonstrated, which will pave the way for the full color display for the PeLEDs.

## Abstract (web summary)

Blue light-emitting diodes (LEDs) are critical for displays. Employing a large organic cation into a quasi-two dimensional perovskite with green emission, Chu et al. achieve LEDs exhibiting a high external quantum efficiency of 12.1% and stable spectra in the sky-blue region.

## Introduction

Lead halide perovskite shows very high potential in display due to its unique emission properties, significant progresses in electroluminescence (EL) for perovskite light-emitting diodes (PeLEDs) have been achieved in recent years^1–10^. The external quantum efficiency (EQE) of red and green emissions have been increased to over than 20%^11–13^, while the efficiency of blue emission is still lag behind because of the difficulties in synthesizing stable materials and maintaining high quantum efficiency in the films^14,15^.

Several attempts have been done to realize blue emission of the PeLEDs. The easiest way is to incorporate chlorine into bromine-based perovskites to tune the bandgap^2,4,16–18^, a 5.7% EQE in blue from Br–Cl halide mixed perovskite has been achieved by Yip et al., recently^14^. However, these Br–Cl mixture perovskite could exhibit unavoidable phase separation when exposed to light or under electrical potential^4,14,19–21^, which further induced peak shift or multi-peaks due to the halide migration under applying voltage. The second approach is by modulating the conduction band via metal ions doping such as Zn^2+^, Cu^2+^, Mn^2+^, Al^3+^ into lead site^22–25^. While the impurity induced by metal elements are considered to be one of the key factors that favors in non-radiative recombination^26^.

To date, the effective attempts to obtain blue PeLEDs are generating the quantum-well structure via reducing-dimensional (quasi-2D and 0D) perovskites with different large cations^7,10,17,27–29^. For example, Sargent et al. have used shorter iso-propylammonium (IPA) molecular to replace long ligands phenylethylammonium (PEA) and tune the quasi-2D PEA_2_A_*n*−1_Pb_*n*_X_3*n*+1_ perovskite composition with a desired *n*, and showed 1.5% EQE in sky blue^30^. During preparation of this manuscript, Jin et al. have reported efficient blue light-emitting diodes (LEDs) based on quantum-confined bromide perovskite via using phenylbutylammonium bromide as quasi-2D phases combined with an anti-solvent film deposition method, a peak EQE of up to 9.5% has been achieved^31^. These strategies are still focus on the modification of the cations in the two dimensional perovskite frame.

In this manuscript, we introduced large cation CH_3_CH_2_NH_2_^+^ (EA) into the Cs^+^ site in PEA_2_(CsPbBr_3_)_2_PbBr_4_ perovskite, and tuned the emission from green (508 nm) into blue (466 nm). The obtained perovskite films showed larger than 70% photoluminescence quantum yield (PLQY) in blue, and more importantly, the blue emission shows good spectra stability under light-soaking and heating. Eventually, by optimizing the content of EABr, a 12.1% EQE of the sky-blue (488 nm) EL was obtained, which could be the highest efficiency for blue emission PeLEDs so far.

## Results

### The thin films characterization

**Fig. 1:**
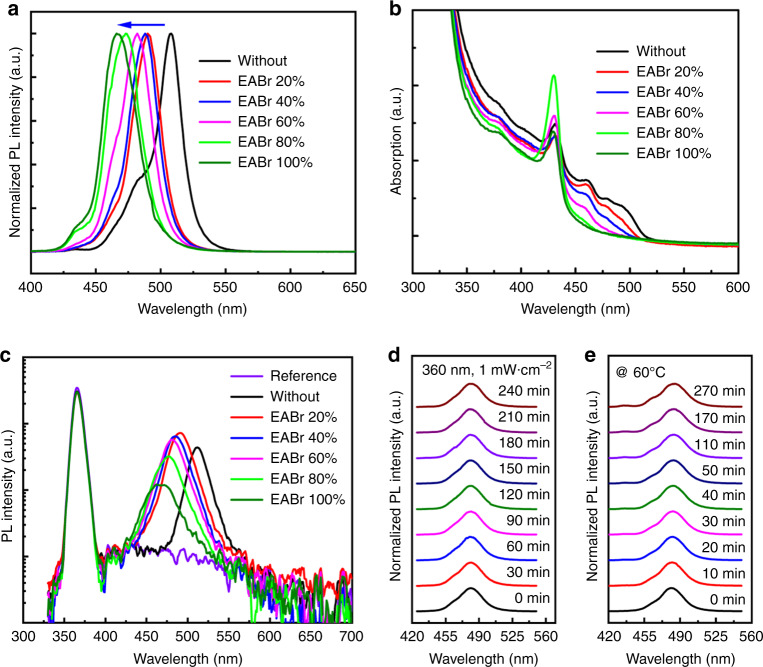
Characteristics of perovskite films with different EABr. **a** Normalized steady photoluminescence (PL) spectra. **b** UV–vis absorption spectra, and **c** Photoluminescence quantum yield (PLQY) spectra. **d** Normalized PL spectra of quasi-2D perovskite film with 60% EABr under continuous UV radiation (360 nm, 1 mW cm^−2^) for different exposure times. **e** Normalized PL spectra of quasi-2D perovskite film with 60% EABr after continuous thermal treatment (60 °C) for different times.

To obtain high efficient blue PeLEDs, quasi-2D perovskite PEA_2_(CsPbBr_3_)_n-1_PbBr_4_ (*n* = 3, see “Methods”) with efficient green emission as the initial composition for modification. The emission wavelength of quasi-2D PEA_2_(CsPbBr_3_)_2_PbBr_4_ was located at 508 nm as shown in Fig. 1a. The three-dimensional perovskite CsPbBr_3_ was chosen as the main core of the emission, excess of CsBr (CsBr:PbBr_2_ = 2:1, molar ratio) has been introduced to passivate the non-radiative defects recombination^9^. The long alkyl chain phenylethylammonium bromide (PEABr) has been generally introduced in the perovskites in order to form a 2D phases when it replaces A-site atoms of the outer, which has been demonstrated to confine the charge and enhance emission^10^.

The quasi-2D PEA_2_(CsPbBr_3_)_2_PbBr_4_ perovskite films with different ratio of EABr were synthesized through mixing PbBr_2_, CsBr, PEABr, and EABr in the solution of dimethyl sulfoxide (DMSO) and a one-step spin-coating process (details in “Methods”). As shown in Fig. 1a, the emission of films can be tuned from 508 nm to 466 nm with increasing EA cations from 0 to 100%. The emission images of these samples under ultraviolet excitation (365 nm) are shown in Supplementary Fig. 1, consistent with PL results, the band edge absorption also showed a blue-shift (Fig. 1b). The PLQY has been increased from 42% for the control film to around 70% while less than 60% is introduced, it was also found that too much EABr (≥80%) lead to the decrease of PLQY (Fig. 1d). The increasing PLQY could be due to the suppression of non-radiative recombination by EABr doping. The reduction of PLQY with the increasing EA cation could be explained that amount of EA has not been introduced into CsPbBr_3_, resulting non-radiative recombination centers. The details in emission properties of these samples are plotted in Supplementary Fig. 2 and summarized in Supplementary Table 1.

The spectra stability is a critical issue for blue emission of perovskite materials. The photo and thermal stability of quasi-2D perovskite film with 60% EABr sealed with 40 mg ml^−1^ poly(methyl methacrylate) (PMMA) as example were studied. The PL spectra of the quasi-2D perovskite with EABr was kept under continuous ultraviolet light illumination (360 nm, 1 mW cm^−2^) for 240 min (Fig. 1d, Supplementary Fig. 3a). The blue perovskite film also illustrates good thermal stability, both of the PL peak position and FWHM were not changed after annealing at 60 °C for 270 min (Fig. 1e, Supplementary Fig. 3b). In comparison, we found the Cl–Br mixed perovskite PEA_2_CsPb(Br_0.1_Cl_0.9_)_3_ PbBr_4_ with emission owns blue region exhibited poor spectral stability (Supplementary Fig. 4).

**Fig. 2:**
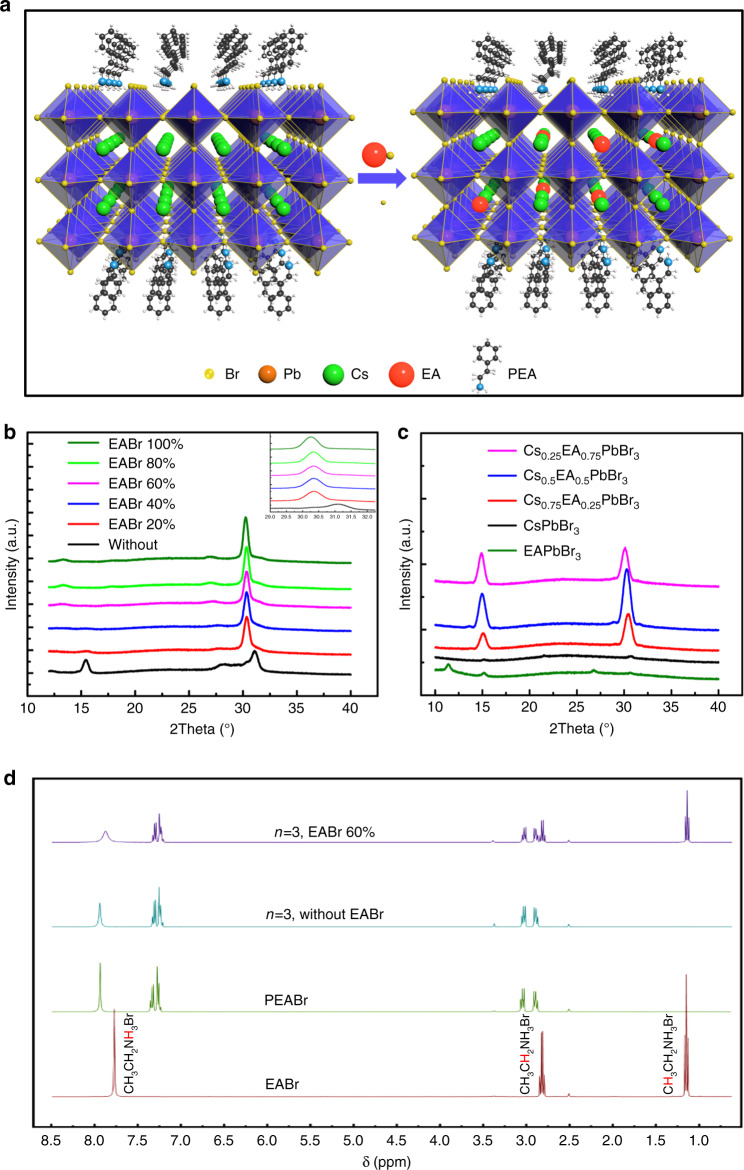
Crystal composition of perovskite films. **a** Schematics showing the EA cation doping in the perovskite lattice to replace Cs^+^ in quasi-2D perovskite. **b** X-ray diffraction (XRD) pattern of the quasi-2D perovskite with different ratio of EABr. The inset is amplified XRD pattern of samples from 29° to 32°. **c** XRD patterns of the Cs_1−*x*_EA_*x*_PbBr_3_ (0 ≤ *x* ≤ 1) perovskite thin films. **d** ^1^H NMR spectra of the EABr, PEABr powers and quasi-2D perovskite with or without EABr.

We carried out the X-ray diffraction (XRD) characterization for PEA_2_(EA_*x*_Cs_1−*x*_PbBr_3_)_2_PbBr_4_ with different EABr to check the phases (Fig. 2b). The XRD patterns of the thin film without EABr shows three main peaks at 15.42°, 30.37°, and 31.03°, which match well with the (100) and (200) planes of CsPbBr_3_ and (006) of Cs_4_PbBr_6_ respectively. It is found that the diffraction peaks from (100) of CsPbBr_3_ and (006) of Cs_4_PbBr_6_ become weaker and finally disappear with increasing EA cation. More importantly, the main diffraction at 30.37° gradually decreased to 30.26°, due to the large molecular size of EA cation (EA^+^, 274 ppm) compared with cesium cation (Cs^+^, 177 ppm)^32^, we can anticipate that the EA cation has been filled into the crystal lattice of CsPbBr_3_ to form Cs_1−*x*_EA_*x*_PbBr_3_ (0 ≤ *x* ≤ 1) perovskite phase in quasi-2D structure (Fig. 2a). To confirm the successful incorporation of EABr in the final quasi-2D perovskite films, we measured the ^1^H NMR spectra of the EABr, PEABr powder and the quasi-2D perovskite films with or without EABr, which were dissolved in DMSO-*d*_6_. The EA signals from EABr power were completely keeping in line with the quasi-2D perovskite with EABr film, as shown in Fig. 2d, which manifested the EA cation can been filled into the quasi-2D perovskite.

To further confirm the insertion of EA in CsPbBr_3_ crystal lattice, the XRD patterns of pure 3D perovskite of CsPbBr_3_ with different EABr were collected (Fig. 2c). Similarly, the introduction of EABr lead to the decrease of the diffraction angles, indicating an expansion of the CsPbBr_3_ crystal lattice by insertion of the larger EA cations. The emission wavelength and optical bandgap of these perovskite films can be tuned from green to blue region by adding EABr into CsPbBr_3_ (Supplementary Fig. 5). It might be argued that a possibility the EA leads to formation of the quasi-2D perovskites by acting as a capping ligand. In that case, we should observe emission or absorption features from two dimensional perovskites in mixed perovskites. However, while the samples with moderate amount of EABr (*x* < 0.75) show only one dominant emission peak and no excitonic absorption peaks at higher energy, providing evidence that these films are only simple phases. Whiling introducing more EABr (*x* ≥ 0.75) in CsPbBr_3_, the films exhibit two photoluminescence peaks and an excitonic absorption, the higher energy emission and sharp absorption peaks is almost overlap with EAPbBr_3_ itself, indicating that EA maybe not completely go into the CsPbBr_3_ while the EA amount is too much. These results are consistent with the results shown in Fig. 1c and could also explain why the PLQY of the PEA_2_(CsPbBr_3_)_2_PbBr_4_ is low while incorporating too much EABr. As we know, the size of guanidinium (GA) molecular is similar as the cation of EA molecular^32^. The GA molecular have been tried to tune the emission of quasi-two dimensional perovskites, unfortunately, it is found that the GA molecular cannot tune the emission from green region into blue (Supplementary Fig. 6).

We carried out morphologies characterization of the perovskite films with different EABr by atomic force microscopy (AFM) and scanning electron microscopy (SEM) (Supplementary Figs. 7 and 8). The root mean square roughness of these films are around 0.5 nm, the smooth film will avoid leakage current. In addition, the crystal size has not been decreased while introducing of the EA into the PEA_2_(CsPbBr_3_)_2_PbBr_4_ perovskite, indicating that the blue shift of the emission could not be originated from quantum confinement effect.

### Density functional theory (DFT) calculation

**Fig. 3:**
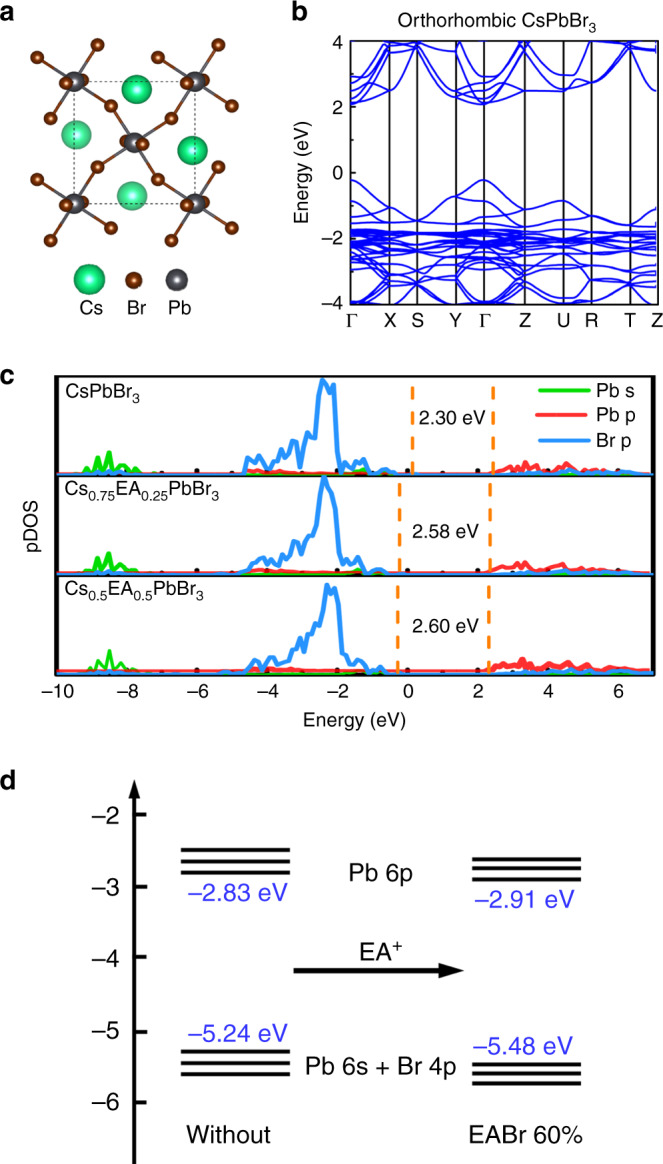
The crystal structures and electronic structures of different perovskites. **a** The crystal structures of CsPbBr_3_. **b** The electronic structures of CsPbBr_3_. **c** The calculated electronic density of states (DOS) of CsPbBr_3_, Cs_0.75_EA_0.25_PbBr_3_, and Cs_0.5_ EA_0.5_PbBr_3_. **d** Schematic representation of variation of energy levels of quasi-2D perovskite in Pb 6p and (Pb 6s + Br 4p) orbitals on insertion of EA cation. The valence band maximum (VBM) and conduction band minimum (CBM) come from (Pb 6s + Br 4p) and Pb 6p orbitals, respectively.

To gain further insights regarding the origin of the band gap increasing in the quasi-2D perovskite, we carried on theoretical band structure calculations within DFT using CsPbBr_3_, Cs_0.75_EA_0.25_PbBr_3_ and Cs_0.5_EA_0.5_PbBr_3_ as the model system. The details are provided in the “Methods” section. A 4 × 3 × 4 k-mesh for orthorhombic unit cell CsPbBr_3_ is performed in relaxation (Fig. 3a). All atomic coordinates and the lattice parameters are relaxed until forces of each atom were less than 0.02 eV. The hybrid functional exchange-correlation functional of Heyd–Scuseria-Ernzerhof (HSE06)^33^ with 25% Hartree-Fock is considered at accurate calculation of electronic structures. The band structure of CsPbBr_3_ is shown in Fig. 2b. The calculated electronic density of states (DOS) of CsPbBr_3_, Cs_0.75_EA_0.25_PbBr_3_, and Cs_0.5_EA_0.5_PbBr_3_ is shown in Fig. 3c, the calculated corresponding bandgaps are 2.30, 2.58, and 2.60 eV, which are in good agreement with the experimental observations in the absorption spectra (2.34, 2.37, and 2.41 eV, Supplementary Fig. 5b).

The first-principle calculation of three-dimensional perovskite has confirmed the increasing of band gap with introduction of EA cation into CsPbBr_3_ lattice. It might be argued that the increase of lattice will generally lead to decrease the bandgap, such as MAPbBr_3_ and FAPbBr_3_ compared with CsPbBr_3_, and why the large size of EA insertion lead to the enlargement of bandgap of CsPbBr_3_? It could be simply explained that the uneven lattice expansion makes the length of the six Pb–Br bonds increase differently, and resulting different decreases for valence band maximum (VBM) and conduction band minimum (CBM) in energy. As we know, the bandgap of the APbBr_3_ is determined by Pb–Br orbitals coupling^34^, the longest bonding Pb–Br length with the smallest coupling will determine the CBM, while the shortest Pb–Br bond with the strongest coupling will control the VBM. The insertion of EA cation could mainly affect the shortest Pb–Br bond which lower the VBM of obviously, and increase the bandgap. More fundamental study of the Cs_1−*x*_EA_*x*_PbBr_3_ system will be carried out to explore the clear reason of the band gap enlargement via EA introduction in the near future. The ultraviolet photoelectron spectroscopy (UPS) results showed that the enlargement of bandgap after introducing EA is mainly due to the downshift of the VBM (Fig. 3d, Supplementary Figs. 9a, b), further confirming the insertion of EA into the CsPbBr_3_ lattice.

## Discussion

Encouraged by above findings, we constructed LEDs using with PEA_2_(Cs_1−*x*_EA_*x*_PbBr_3_)_2_PbBr_4_ as active layer. A device structure glass/ITO/m-PEDOT:PSS (35 nm)/Perovskite (25 nm)/TBPi (40 nm)/LiF (1 nm)/Al (70 nm) is adopted, in which m-PEDOT:PSS is PSS-Na modified PEDOT:PSS to increase the work function forming better level alignment for hole injection and electron blocking^10,35^. The schematic of the band alignment diagram and the cross-section SEM image of the completed device are shown in Supplementary Fig. 10.

**Fig. 4:**
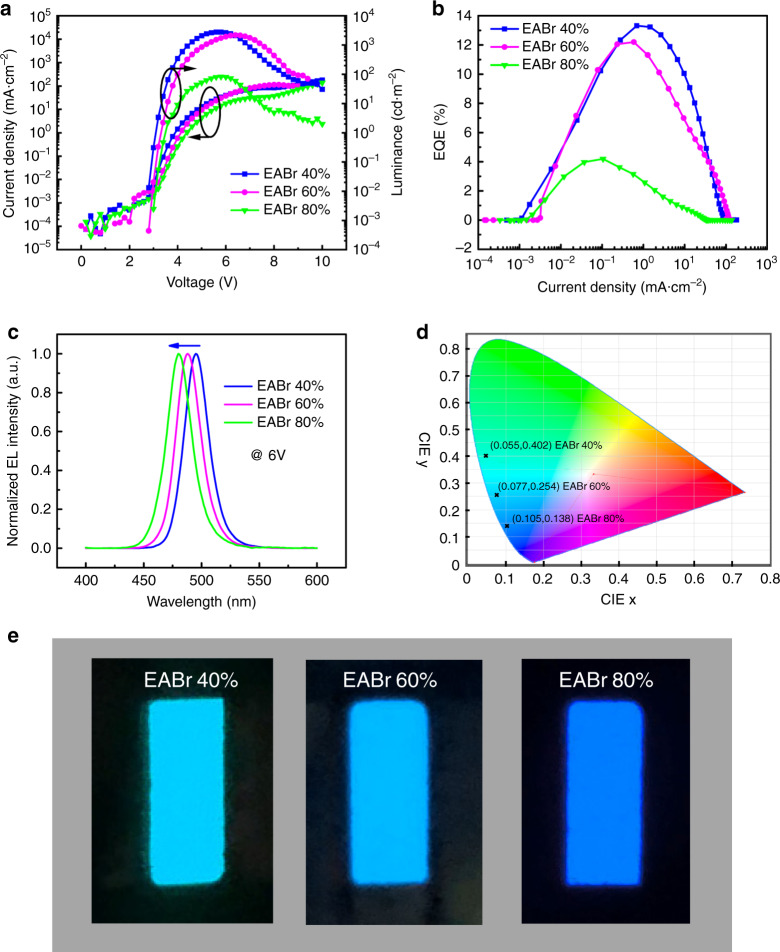
Performance of the PeLEDs with different ratio of EABr. **a** Characterization of current density and luminance versus applied voltage. **b** Characterization of external quantum efficiency (EQE) versus current density. **c** Normalized electroluminescence (EL) spectra of perovskite LEDs with 60% EABr under increasing applied voltage. **d** Commission Internationale de l’Eclairage (CIE) values of the EL spectra of perovskite LEDs. **e** Electroluminescence images of the perovskite LEDs with different EABr.

The performance of the PeLEDs are characterized and summarized in Fig. 4a–c and Table 1. It can be found that the maximum brightness are 2790, 2191, and 83 cd m^−2^ (Fig. 4a), the EQE are 13.3%, 12.1% and 4.19% (Fig. 4b) and the EL are located at 495, 488 and 480 nm (Fig. 4c) for 40%, 60% and 80% EABr, respectively. The normalized EL spectra of PeLEDs with different ratios of EABr was obtained at 6 V and all the emissions held up narrow full-widths at half-maximum (FWHMs) of ≤25 nm, resulting in the high color purity in Commission Internationale de l’Eclairage (CIE) coordinate as shown in Fig. 4e. It is obvious that the EL image of varying EABr was in line with the result of EL spectra and CIE (Fig. 4f). With the blue shift of EL spectra, the corresponding CIE value and the EL image of different EABr also changed from the green to blue region, indicating the successful achievement of a blue-emissive perovskite LED. To our best knowledge, the 12.1% EQE for sky-blue emission at 488 nm could be the best for the PeLEDs. The lower efficiency while shifting the emission into deeper blue region could be due to the low PLQY and also large poor injection (Figs. 1c and 4a). A histogram of peak EQEs for 28 devices shows an average value of ~9.5% in Supplementary Fig. 11, the statistical data mean that the performance of PeLEDs device owns good reproducibility.

**Table 1:** Summary of quasi-2D perovskite LEDs with different ratios of EABr.

EABr ratio	Max. EQE (%)	Max. CE (cd·A^−1^)	Max. L. (cd·m^−2^)	EL peak (nm)	FWHM (nm)	CIE
40%	13.3	24.45	2790	495	23	(0.055, 0.402)
60%	12.1	17.58	2191	488	25	(0.077, 0.254)
80%	4.19	6.17	83	480	25	(0.105, 0.138)

*LED* light-emitting diode, *EABr* ethylammonium bromide, *EQE* external quantum efficiency, *CE* current efficiency, *L.* luminance, *EL* electroluminescence, *FWHM* full-width at half-maximum, *CIE* Commission Internationale de I’Eclairage.

The spectra stability of EL under applied voltage was tested, it is clear that the normalized EL spectra of PeLEDs with 60% EABr is unchanged with the increasing applied voltage (Fig. 4d), suggesting that the emission spectra is stable under applied voltage. We have collected about 12 min video of our LEDs working at a constant current density of 0.3 mA cm^−2^, it can be found that EL show the constant color at varying the time (Supplementary Movie 1). In comparison, as similar as photoluminescence, the EL spectra changed for mixed halide perovskite with increasing the voltage (Supplementary Fig. 12). We have carried out the device stability test at a constant current density of 1.5 mA cm^−2^ and also at the 100 cd m^−2^, we found that the device without encapsulation could survive around 1 h in nitrogen glove box (Supplementary Fig. 13). The increasing input voltage at constant current brings increasing input power, suggesting that increasing Joule heating takes place in the device, which could contribute for instability of PeLEDs.

In conclusion, we have demonstrated that the blue emission of perovskite LEDs with EA cation incorporation into three-dimensional perovskite lattice. The emission has been tuned from green into blue, and showed high PLQY (>70%). In addition, the emission spectra is stable under photo and thermal stress. With tailoring the EA composition, an efficient PeLEDs with 12.1% EQE in 488 nm emission has been achieved. This concept is expected to open an avenue for full color display using PeLEDs.

## Methods

### Materials

CsBr, PbBr_2_, DMSO and poly (sodium 4-styrenesulfonate) (PSS-Na, average Mw ~ 70,000) were purchased from Sigma-Aldrich. 2,2’,2”-(1,3,5-Benzinetriyl)-tris(1-phenyl-1-H-benzimidalzole) (TBPi), and ethylam-momium bromide (EABr) were purchased from Xi’an Polymer Technology Corp. PEABr was purchased from Dyesol (now Greatcell Solar). The modified PEDOT:PSS (m-PEDOT:PSS) solution is mix of normal PEDOT:PSS (AI 4083) aqueous solution and 100 mg ml^−1^ PSS-Na aqueous solution by a volume ration of 3:1.

### Perovskite solution preparation

The Cs_*x*_EA_1−*x*_PbBr_3_ and PEA_2_PbBr_4_ precursor solution were precursor solution were prepared by dissolving appropriately stoichiometric CsBr, EABr, PEABr, and PbBr_2_ in DMSO under continuous stirring for 4 h at room temperature, keeping the molar concentration PbBr_2_ at 0.1 M. The CsPbBr_3_ precursor solution was also prepared by dissolving CsBr and PbBr_2_ (2:1 in molar) in DMSO under continuous stirring for 4 h at room temperature, keeping Pb^2+^ at 0.1 M, too. The quasi-2D perovskite PEA_2_(CsPbBr_3_)_2_PbBr_4_ precursor solution was mixed the PEA_2_PbBr_4_ and CsPbBr_3_ by a volume ration of 1:2. The ratio of m% EABr refers to the molar between EABr and PbBr_2_.

### Device fabrication

The indium tin oxide (ITO)-coated glass substrates were sequentially cleaned by sonication in detergent, deionized water, acetone and isopropyl alcohol and then dried by N_2_. After a ultraviolet ozone treatment for ITO in 10 min, the modified PEDOT:PSS aqueous solution was spin-coated onto the ITO substrate at 9000 rpm for 60 s and baked at 160 °C for 15 min in ambient air. Thereafter, the substrates were transferred into a nitrogen-filled glove box, and the perovskite precursor solution was then spin-coated on the m-PEDOT:PSS film at 3000 rpm for 2 min, followed by annealing on a hot plate at 60 °C for 20 min. Finally, the fabrication of PeLEDs was completed by depositing TBPi (40 nm) and LiF/Al electrodes (1 nm/100 nm) layer by layer through a shadow mask in high vacuum thermal evaporator. The device area was 10 mm^2^ as defined by overlapping area of ITO and Al electrode.

### First-principle calculations

The electronic structures of CsPbBr_3,_ Cs_0.75_EA_0.25_PbBr_3_, and Cs_0.5_EA_0.5_PbBr_3_ are calculated using DFT methods (employing the ab initio code VASP)^36^ with the projector-augmented wave (PAW) pseudopotentials^37^. A plane wave cutoff energy of 400 eV and the Perdew-Burke-Ernzerhof revised for solids (PBEsol) exchange-correlation functional are employed^38^.

### Perovskite film and device characterizations

The SEM images were obtained using a field-emission SEM (FEI NanoSEM650), which used an electron beam accelerated at 500 V to 30 kV, enabling operation at a variety of currents. AFM measurements were carried out using a Bruker FASTSCANBIO in non-contact mode. The XRD patterns of the perovskite thin films were performed with a Rigaku D/max 2500H equipment with a conventional Cu target X-ray tube (Cu K-alpha, *λ* = 1.5405 Å) as the X-ray source. Scans were taken with a 0.5-mm-wide source and detector slits, with X-ray generator settings at 40 kV and 30 mA. Proton nuclear magnetic resonance (^1^H NMR) spectra were recorded on Bruker Avance II 400 MHz system with BBI probe. Steady-state PL spectra of the perovskite films was measured at room temperature in the ambient air using a FLS1000 spectrometer. Absorption spectra were acquired using an ultraviolet-visible spectrometer (Cary 5000). PLQYs of the perovskite thin films were recorded by a commercialized PLQY measurement system (LQE-50-PL) from Enlitech with excitation from a 368 nm LED. UPS spectra was performed on a Thermo Scientific ESCALab250Xi with an applied bias of −10 V. The He I emission line at 21.22 eV was employed. The Helium pressure in the analysis chamber during measurement was about 3 × 10^−8^ mbar. The film samples over the ITO layer had a conductive connection with an Au sample, so the Fermi level value of the film samples is equal to that of Au samples. The work function *Ф* (that is the Fermi level absolute value of the free film) of the test films can be calculated from following equation: *hν* – *Ф* = *E*_Fermi_ – *E*_cutoff_, where *E*_Fermi_ and *E*_cutoff_ is the value of Fermi level position and the steep edge position in UPS spectrum, respectively, *hν* = 21.22 eV and *E*_Fermi_ = 21.51 eV. The I-V-L curve, EL spectrum, EQE, CIE, and operating lifetime of the perovskite LED were carried out simultaneously by a commercialized system (LQE-50-EL, Enlitech) that was equipped with an integrated sphere and photomultiplier tubes (PTM), in which the PMT is used to measure the low luminance. All the device characterization tests of perovskite LEDs were recorded at room temperature in the ambient air for the un-encapsulated devices, except that the operational stability test was carried out in an N_2_-filled glovebox. To confirm our measurement results, cross-checking with other research groups was carried out. We tried our best to encapsulate the devices and measured at the different research units, and found that the measurement results are almost consistent. Some of the devices showed about 10% difference, which could be due to the degradation during the transmission.

## Supplementary information

Supplementary Information

Description of Additional Supplementary Files

Supplementary Movie 1

## Data Availability

The data that support the findings of this study are available from the corresponding author upon request.
