# Supplementary material for: Large cation ethylammonium incorporated perovskite for efficient and spectra stable blue light-emitting diodes
Source: Nat Commun. 2020 Aug 20;11:4165. doi: 10.1038/s41467-020-17943-6 (PMC7441179; doi:10.1038/s41467-020-17943-6)
Supplement: Supplementary file 1 — Supplementary Information [file 41467_2020_17943_MOESM1_ESM.pdf]

Supplementary Information

Large cation ethylammonium incorporated perovskite for efficient and spectra stable blue light-emitting diodes

Zema Chu[1, 2], Yang Zhao[1, 2], Fei Ma[1, 2], Cai-xin Zhang[2, 3], Huixiong Deng[2, 3], Feng Gao[1, 2], Qiufeng Ye[1, 2], Junhua Meng[1, 2], Zhigang Yin[1, 2], Xingwang Zhang[1, 2*] and Jingbi You[1, 2*]

[1]Key Laboratory of Semiconductor Materials Science, Institute of Semiconductors, Chinese Academy of Sciences, Beijing, P. R. China. 100083, [2]Center of Materials Science and Optoelectronics Engineering, University of Chinese Academy of Sciences, Beijing, P. R. China. 100049. [3]State Key Lab Superlattices & Microstruct, Beijing 100083, Institute of Semiconductors, Chinese Academy of Sciences, Beijing, P. R. China. 100083. [*]Corresponding author. E-mail: xwzhang@semi.ac.cn (X. Z.) or jyou@semi.ac.cn (J. Y.)

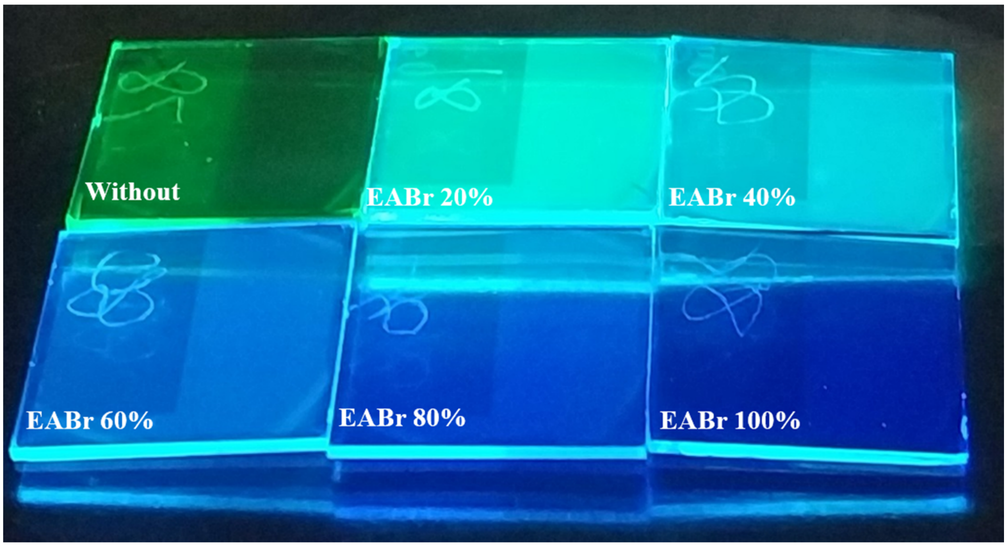

Supplementary Figure 1| The emission images of perovskite films with different ratios of EABr under ultraviolet excitation (365 nm).

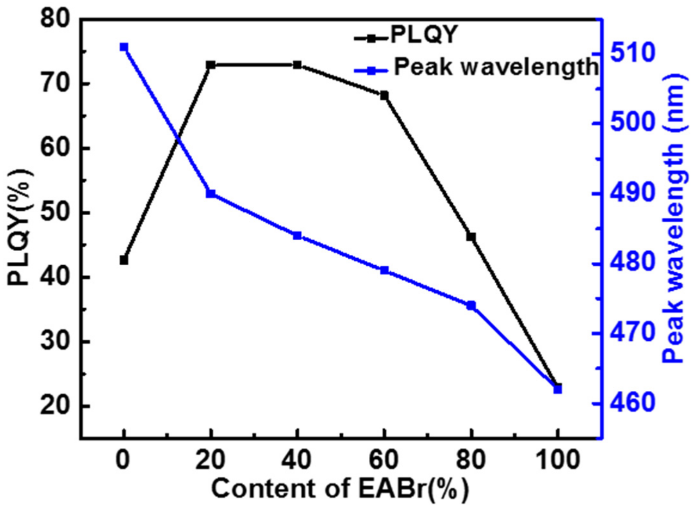

Supplementary Figure 2| The photoluminescence quantum yield (PLQY) and the emission peak wavelength of perovskite films with different ratios EABr.

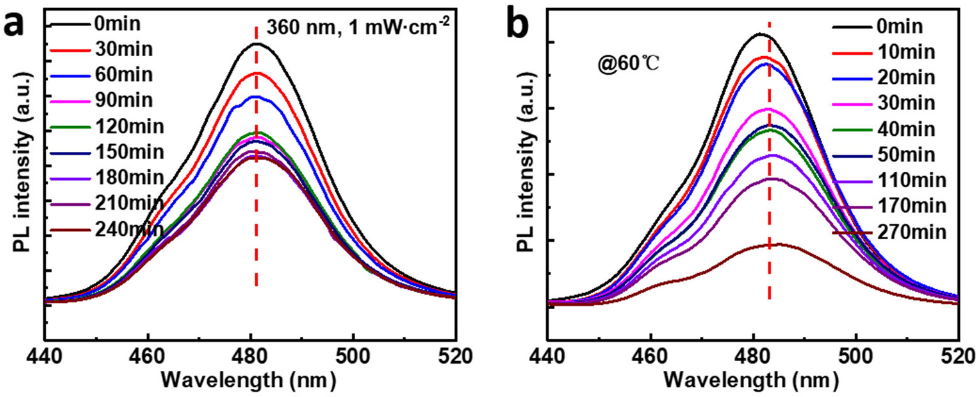

Supplementary Figure 3| The spectra stability of quasi-2D perovskite films with 60 % EABr. **a,** PL spectra of perovskite films under continuous UV radiation (360 nm, 1 mW • cm^{-2}) for different exposure times. **b,** PL spectra of perovskite films after continuous thermal treatment (60 °C) for different times.

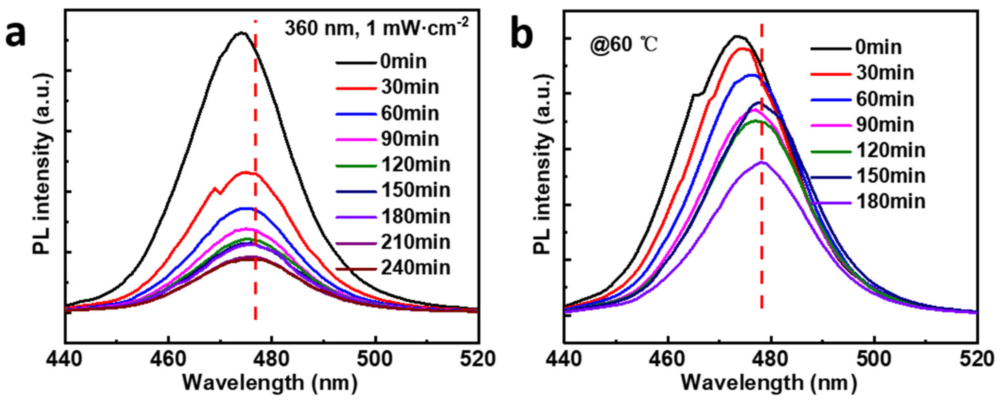

Supplementary Figure 4| The spectra stability of the $PEA_2PbBr_4CsPb(Br_{0.1}Cl_{0.9})_3$ films. **a**, The PL spectral redshift from 474 nm to 477 nm under UV light soaking for 240 min. **b**, The PL spectral redshift from 474 nm to 478 nm after annealing at 60 ºC for 180 min.

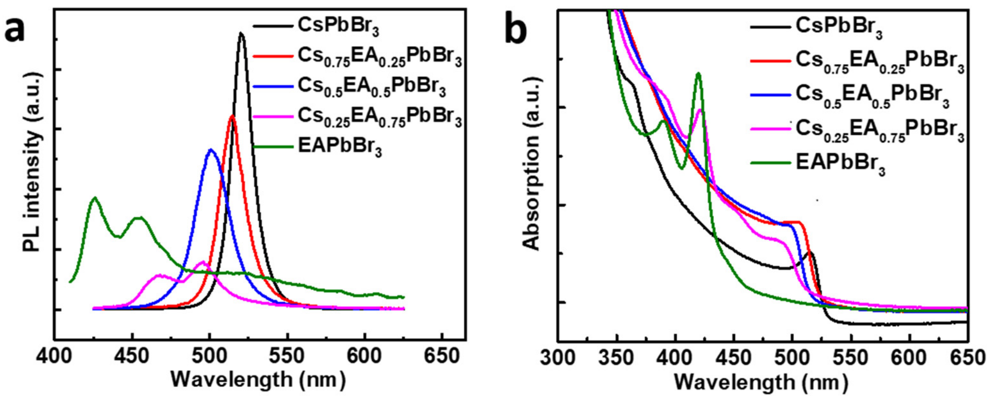

Supplementary Figure 5| Characteristics of $Cs_{1-x}EA_xPbBr_3$ (x= 0, 0.25, 0.5, 0.75 and 1) films. **a,** Steady-state PL spectra of $Cs_{1-x}EA_xPbBr_3$ films. **b,** UV-vis absorption of $Cs_{1-x}EA_xPbBr_3$ films.

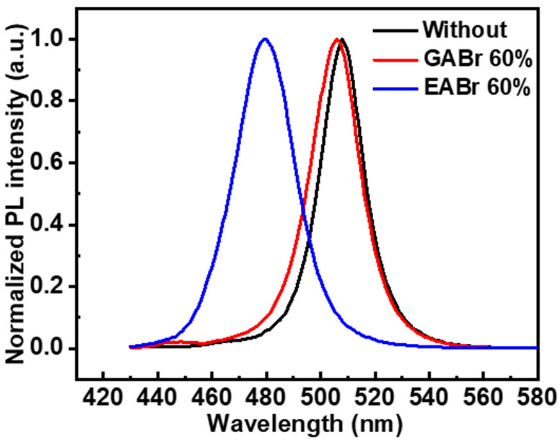

Supplementary Figure 6| The PL measurement of the quasi-2D perovskite film without or with 60% EABr or 60% GABr.

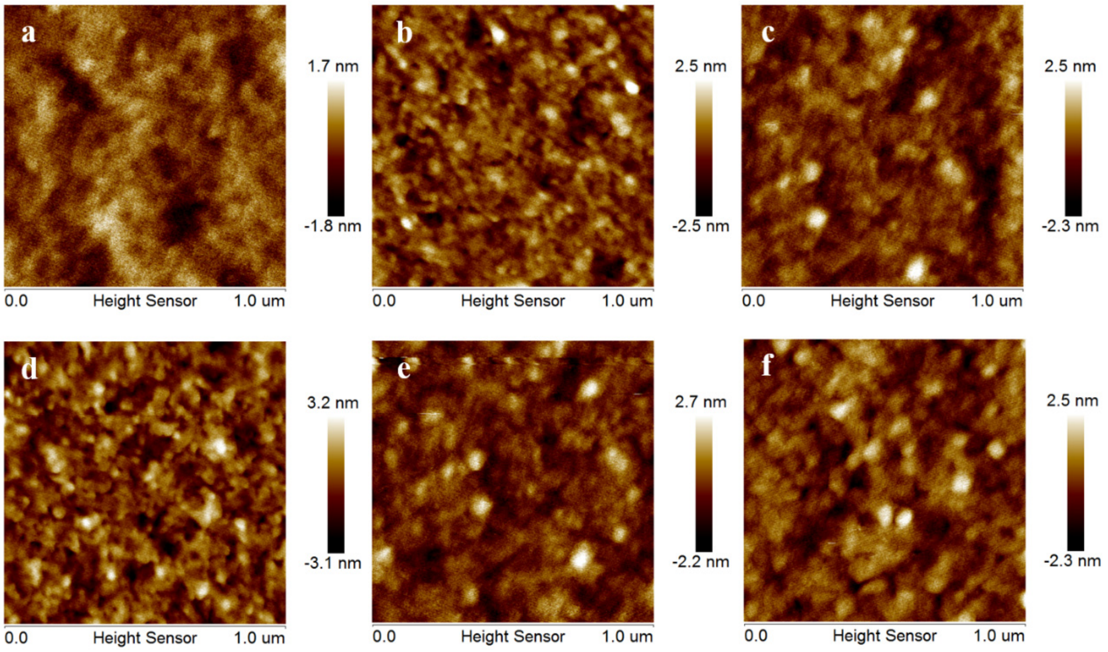

Supplementary Figure 7| The atomic force microscopy images of quasi-2D perovskite films with different ratio EABr. a, 0%. b, 20%. c, 40%. d, 60%. e, 80%. and f, 100%.

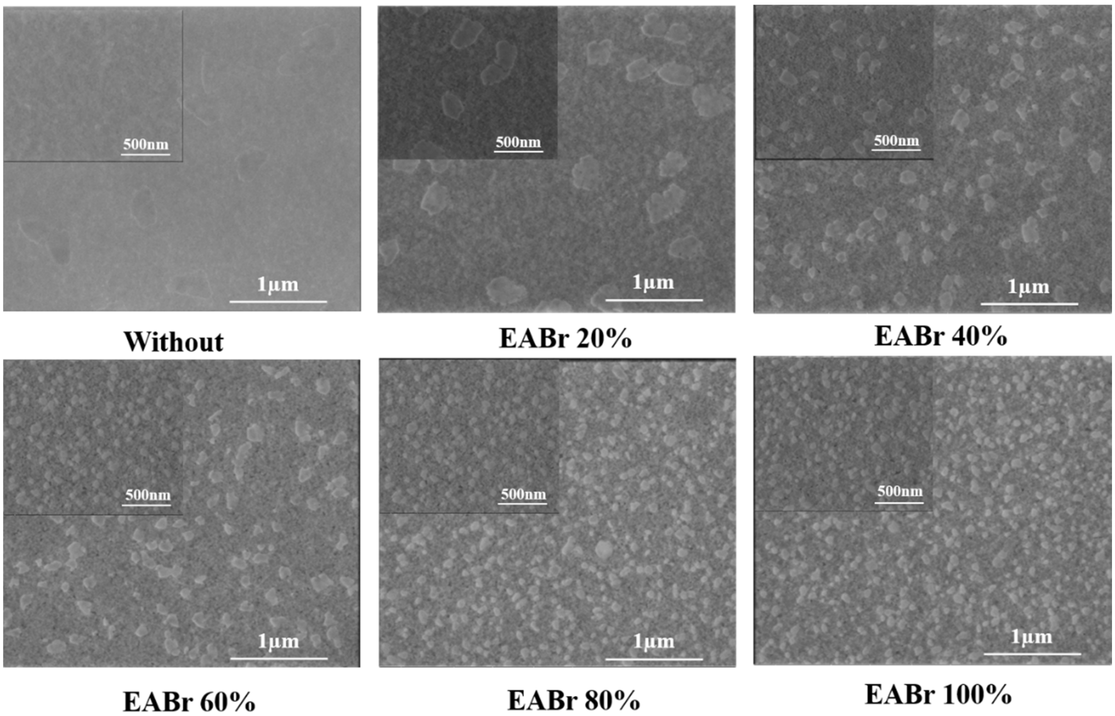

Supplementary Figure 8| The scanning electron microscopy of quasi-2D perovskite films with different ratios of EABr.

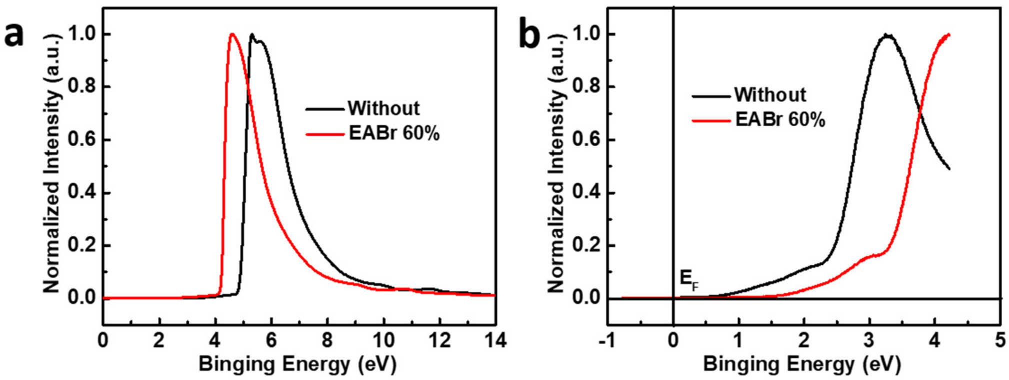

Supplementary Figure 9| Band structure properties of the perovskite films with varying amount of EABr (without and 60%). **a,** Ultraviolet photoelectron spectroscopy (UPS) cutoff edge of perovskite films. **b,** Valence band spectra of perovskite films from UPS measurements.

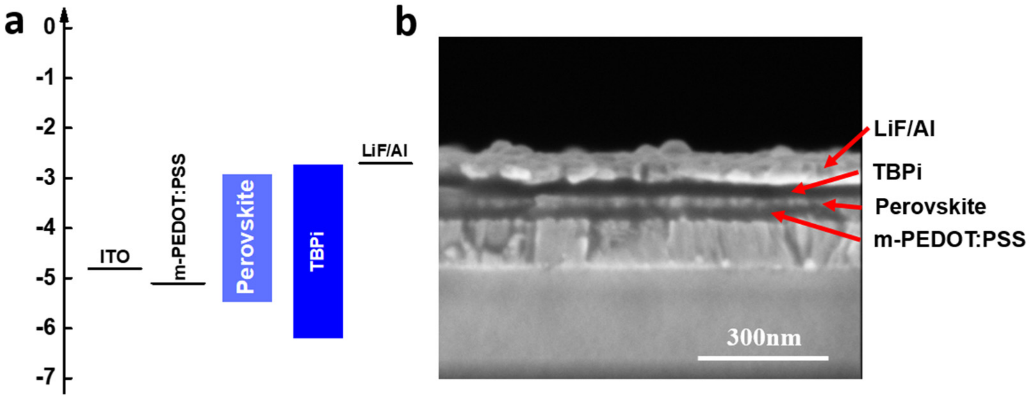

Supplementary Figure 10| The structure of the completed device. **a,** The band alignment diagram of the devices. **b,** The cross-section SEM image of the device.

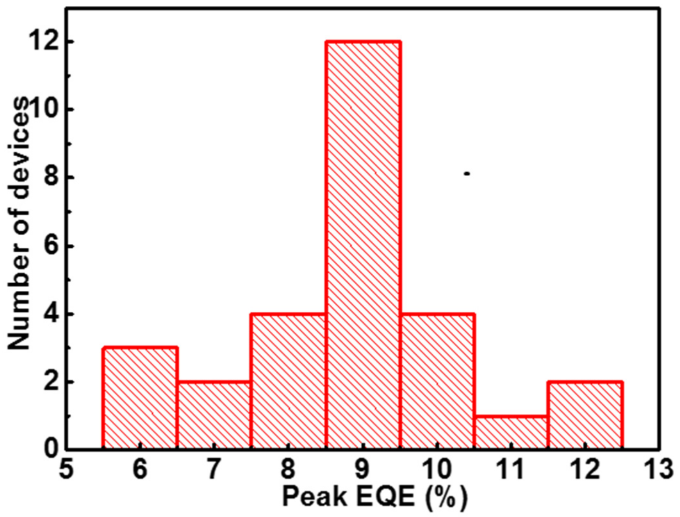

Supplementary Figure 11| A histogram of peak EQEs from 28 devices.

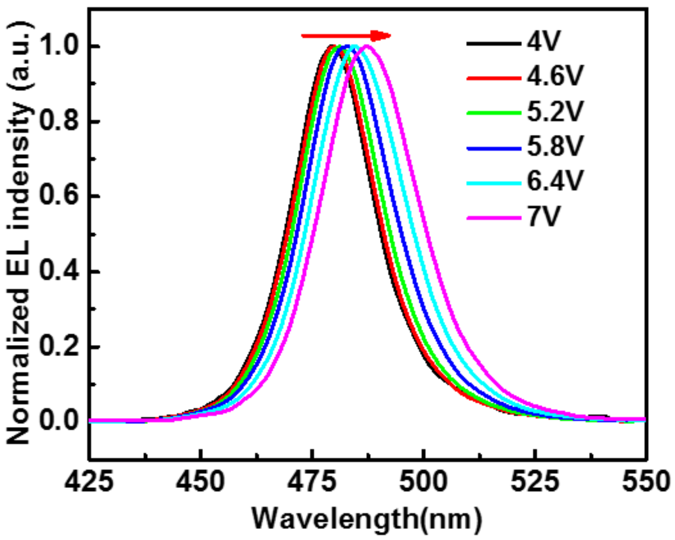

Supplementary Figure 12| The electroluminescence spectra of mixed-halide perovskite ($PEA_2PbBr_4{\cdot}CsPb(Br_{0.1}Cl_{0.9})_3$) LEDs with increasing applied voltage.

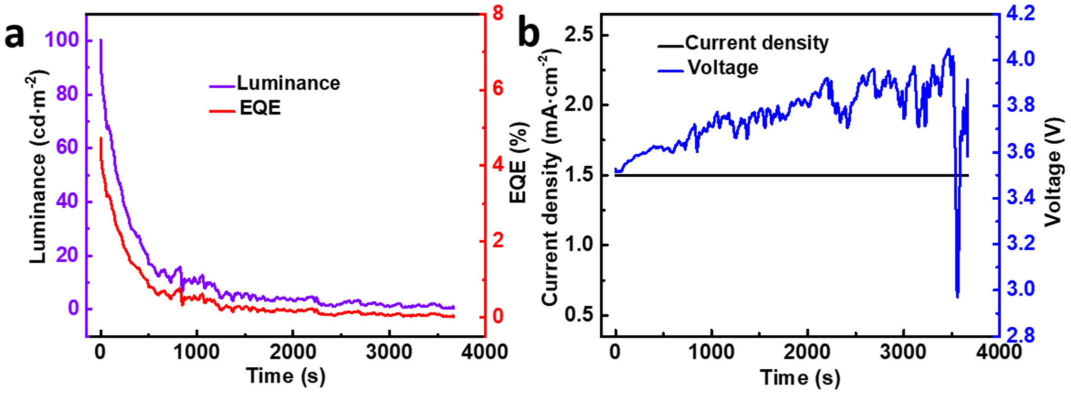

Supplementary Figure 13| a, The stability of the PeLEDs without encapsulation under a constant injection current (1.5 mAcm^{-2}) in nitrogen filled glove box. **b**, The variation of input voltage with the operation time.

Supplementary Table 1| Summary of the emission properties of $PEA_2(EA_xCs_{1-x}PbBr_3)_2PbBr_4$ quasi-2D perovskite with different ratios of EABr.

**EABr ratio**	**PL peak (nm)**	**FWHM (nm)**	**QY (%)**
**0%**	508	22	42.64
**20%**	490	26	72.85
**40%**	488	26	72.90
**60%**	481	30	68.17
**80%**	473	30	46.26
**100%**	466	30	22.85
