# Supplementary material for: Large cation ethylammonium incorporated perovskite for efficient and spectra stable blue light-emitting diodes
Source: Nat Commun. 2020 Aug 20;11:4165. doi: 10.1038/s41467-020-17943-6 (PMC7441179; doi:10.1038/s41467-020-17943-6)
Supplement: Supplementary file 2 — Description of Additional Supplementary Files [file 41467_2020_17943_MOESM2_ESM.pdf]

Description of Additional Supplementary Files

File Name:Supplementary Movie 1
Description: The video shows the electroluminescence emission of our LED at a constant current density of 0.3 $mA \cdot cm^{-2}$ for about 12 minutes. The emission color is constant, the spectra is stable from the electroluminescence results.
